# Early bleb parameters as long-term prognostic factors for surgical success: a retrospective observational study using three-dimensional anterior-segment optical coherence tomography

**DOI:** 10.1186/s12886-019-1159-1

**Published:** 2019-07-19

**Authors:** Utako Tsutsumi-Kuroda, Sachi Kojima, Ayako Fukushima, Kei-Ichi Nakashima, Keiichiro Iwao, Hidenobu Tanihara, Toshihiro Inoue

**Affiliations:** Department of Ophthalmology, Faculty of Life Sciences, Kumamoto University, 1-1-1 Honjo, Chuo-ku, Kumamoto, 860-8556 Japan

**Keywords:** Glaucoma, Trabeculectomy, Anterior segment OCT, Filtration opening, Surgical success

## Abstract

**Background:**

The object of this study is to investigate the effect of early bleb parameters measured by three-dimensional anterior-segment optical coherence tomography on the surgical success of trabeculectomy.

**Methods:**

This retrospective study included 45 patients with 19 of exfoliation glaucoma, 17 of primary open angle glaucoma, 4 of neovascular glaucoma, 4 of uveitic glaucoma and 1 of glaucoma caused from familial amyloid polyneuropathy who underwent trabeculectomy. Bleb parameters, such as total bleb height, the position and the width of filtration openings on the scleral flap, bleb wall thickness, fluid-filled cavity height, and bleb wall intensity were assessed by three-dimensional anterior-segment optical coherence tomography 0.5 months after trabeculectomy, and were subjected to a Cox proportional hazard model as potential prognostic factors. Surgical success was defined as: IOP < 21 mmHg (A), < 18 mmHg (B), < 15 mmHg (C) with (qualified success) or without medication (complete success). Complete failure was defined as hypotony and additional glaucoma surgeries required.

**Results:**

The width of filtration openings was identified as a prognostic factor for all criteria. By multivariable analysis, the width of the filtration openings was a prognostic factor in all criteria tested, and the preoperative IOP were significant prognostic factors for surgical success in qualified success in criteria B and C. Separate from the median widths of filtration openings, wide filtration opening showed significant survival ratio for qualified success in criteria A and B and for complete success in all criteria, respectively.

**Conclusions:**

The width of filtration opening at an early stage is a prognostic factor for surgical success of trabeculectomy.

## Background

Trabeculectomy is one of the standard surgical modalities worldwide for glaucoma to prevent the progression of visual loss by lowering intraocular pressure (IOP). After a trabeculectomy, aqueous humor flows into the filtering bleb, consisting of the fluid-filled cavity, the scleral flap, and the wall of conjunctival tissue, and is then absorbed into surrounding tissues. Because the IOP-lowering effect of a trabeculectomy depends on the outflow volume of aqueous humor through the filtering bleb, management of the bleb is a key for surgical success. The function of the bleb is related to its morphology and its internal structures; thus, filtering blebs have been observed clinically by various imaging methods, such as slit-lamp examination, photography, ultrasound biomicroscopy (UBM), and optical coherence tomography (OCT) [1–9]. Among the OCT devices, three-dimensional anterior segment OCT (3D AS-OCT) can be useful to identify the aqueous route after a trabeculectomy and exploring the bleb morphology (internal and external) [10, 11].

Our previous studies revealed that the width of the filtration openings on the scleral flap, detectable using 3D AS-OCT, decreased in a time-dependent manner and that the width at an early stage correlated with future IOP [12, 13]. Thus, early bleb parameters may be potential prognostic factors for surgical success after trabeculectomy. However, whether these parameters actually affect the success after trabeculectomy, according to various criteria, remains unclear. Here, we report the predictive value of the width of the filtration openings on surgical results in patients with open angle glaucoma using a Cox proportional hazard model.

## Methods

The Institutional Review Board of Kumamoto University approved the present study. Every investigation adhered to the tenets of the Declaration of Helsinki. Included were patients who received trabeculectomy with mitomycin C between January 2012 and October 2012 at the Kumamoto University Hospital, with analyzable 3D AS-OCT (Casia; Tomey, Nagoya, Japan) images of bleb 2 weeks after trabeculectomy, and be followed more than 1 year. Patients who had undergone combined surgery of trabeculectomy and phacoemulsification were excluded. Data were collected from medical records. When both eyes in one patient met the criteria, one eye treated first was included in this analysis. Prior to analyses, all patient data were anonymized by an independent person of the authors.

Trabeculectomy and postoperative management were performed as described previously [12]. Briefly, a limbus-based or fornix-based conjunctival flap was made, after which a single triangle scleral flap was made. Exposed tissues were treated with 0.04% mitomycin C for 4 min, and subsequently the tissues were washed by balanced salt solution (200 mL). Then, a trabecular block and peripheral iris were excised. Finally, the scleral flap and conjunctival flap were sutured by 10–0 nylon. In all cases, antibiotic eye drop and betamethasone eye drop were used 4 times a day for 3 months.

**Fig. 1:**
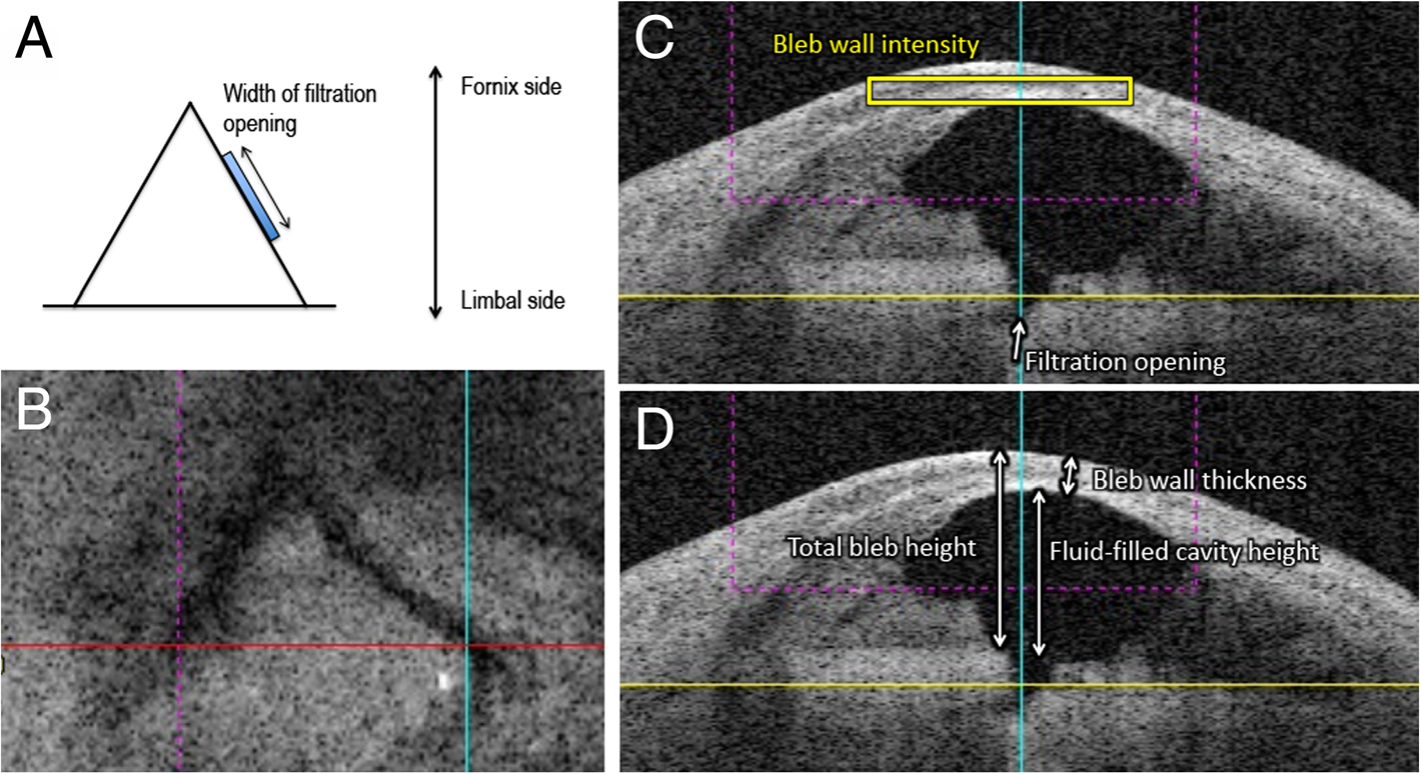
**a** Schematic description of a triangle scleral flap in the filtering bleb, and the width of filtration opening (blue box). **b-d**. An OCT image of the filtering bleb in the C-scan image showing a triangle scleral flap (**b**), and in the horizontal image (**c**, **d**). The red and blue lines indicate the horizontal and vertical axes, respectively. The yellow line represents the z-axis for C-scan image (**b**). The cross points of the colored lines indicate filtration opening

We evaluated OCT images using CASIA Bleb Assessment Software, ver. 4.0 L, (Tomey), as described previously [12, 13]. Briefly, consecutive filtration openings (pits and/or troughs in the fluid-filled cavities) on the scleral flap were examined, and width of the filtration opening was determined. After identification of the filtration openings, reviewers measured the total bleb height, fluid-filled cavity height, bleb wall thickness, and bleb wall intensity in the horizontal image at the center of the filtration opening, or at the center of the scleral flap when the filtration opening was unidentifiable (Fig. 1). Bleb wall intensity was automatically calculated as a mean of optical intensity in the selected area by a built-in software. The mean values of the bleb parameters from three independent reviewers were utilized for further analysis.

We defined surgical success along the World glaucoma Association guidelines as described previously [14]. Briefly, an IOP of < 21 mmHg (criterion A), < 18 mmHg (criterion B), or < 15 mmHg (criterion C) with (qualified success) or without (complete success) the use of topical ocular hypotensive medication(s). Follow up was stopped when any additional non-glaucoma related surgery was performed. We defined complete failure as the requirement for additional glaucoma surgeries including needle bleb revision and hypotony less than 4 mmHg. The IOPs obtained at 2 months or more after trabeculectomy were utilized to avoid any effect of short-term postoperative IOP fluctuations for this definition of surgical success. We used Kaplan-Meier survival-curve analysis and the Cox proportional hazards test to evaluate the cumulative success probability and to estimate the effect of prognostic factors in the univariable analysis. We analyzed the following variables as potential prognostic factors for surgical success: gender, age at the time of the trabeculectomy, history of cataract surgery, preoperative IOP value, design of scleral flap, and 3D AS-OCT parameters. Potential prognostic factors for surgical success (*P* < 0.10 by univariable analysis) were assessed using subsequent multivariate analysis. We analyzed data by the JMP (ver. 10) statistical package (SAS Institute, Cary, NC). A probability (P) value of < 0.05 was considered to indicate statistical significance.

## Results

In total, 52 eyes (52 patients) were enrolled, and 45 eyes completed more than 1-year of follow-up (Table 1). Seven eyes (15.6%) were excluded from the analysis because of loss to follow-up or failure to acquire reliable 3D AC-OCT images during the observational period. In 45 eyes, needling bleb revisions for 3 eyes, repeat trabeculectomy for 7 eyes, additional glaucoma drainage-device surgery for 4 eyes, a conjunctival bleb reconstruction for 1 eye, cataract surgery for 7 eyes and an intraocular lens repositioning for 1 eye were conducted. Two eyes were considered surgical failures due to postoperative hypotony.

**Table 1:** Demographic characteristics of patients

Number of eyes	45
Mean age ± SD (years)	64.3 ± 11.7
Gender (male/female)	33/12
Mean follow-up period ± SD (months)	37.0 ± 15.9
Design of conjunctival flap (FB/LB)	26/19
Mean IOP ± SD (mmHg)	28.7 ± 8.2
Cause of glaucoma	
EXG (%)	19 (42.0)
POAG (%)	17 (37.8)
NVG (%)	4 (8.9)
UG (%)	4 (8.9)
FAP (%)	1 (2.2)
History of prior intraocular surgery	
Cataract surgery (%)	17 (37.8)
Trabeculectomy (fornix-based) (%)	7 (15.6)
Vitrectomy (%)	5 (11.1)

*EXG* exfoliation glaucoma, *FAP* familial amyloid polyneuropathy, *FB* fornix-based, *IOP* intraocular pressure, *LB* limbal-based, *NVG* neovascular glaucoma, *POAG* primary open angle glaucoma, *SD* standard deviation, *UG* uveitic glaucoma

In the Cox proportional hazards test, univariate analysis indicated that the width of the filtration openings was a significant prognostic factor for surgical success of the trabeculectomy (Table 2). The lower preoperative IOP had a potential to relate to qualified success in criteria B and C and for complete success in criterion B (*P* < 0.10). Thus, we assessed influence of width of filtration opening and preoperative IOP by multivariate analysis. The width of the filtration openings was a prognostic factor in all criteria tested, and the lower preoperative IOP were significant prognostic factors for qualified success in criteria B and C (Table 3).

**Table 2:** *P* values calculated by univariable analysis using a Cox proportional hazards model for the surgical success

Parameter	Complete success^a^			Qualified success^a^		
	< 15 mmHg	< 18 mmHg	< 21 mmHg	< 15 mmHg	< 18 mmHg	< 21 mmHg
Total bleb height	0.8017	0.6070	0.6100	0.5359	0.6149	0.3480
Fluid-filled cavity height	0.2553	0.7886	0.9244	0.1331	0.7217	0.7299
Bleb wall thickness	0.6797	0.6182	0.4898	0.1281	0.6871	0.4319
Bleb wall intensity	0.4804	0.8153	0.9750	0.1281	0.5751	0.7725
Distance from scleral-flap top to filtration opening	0.3486	0.1339	0.1965	0.1870	0.1741	0.1057
Width of filtration openings	0.0143*	0.0272^*^	0.0149^*^	0.0029**	0.0410*	0.0465^*^
Age	0.6705	0.8872	0.8876	0.5536	0.8018	0.7674
Male	0.5002	0.9088	0.9285	0.7957	0.8828	0.8546
Preoperative IOP	0.1032	0.0934	0.1275	0.0763	0.0725	0.1783
Pseudophakia	0.1956	0.5105	0.3705	0.9507	0.5347	0.7422
Design of scleral flap (LB/FB)	0.2807	0.6126	0.4688	0.8881	0.5872	0.5499

^a^Surgical failure was defined as an IOP value ≥21 mmHg, ≥ 18 mmHg, or ≥ 15 mmHg with (qualified success) or without (complete success) the use of topical ocular hypotensive medication(s). Complete failure was defined as additional glaucoma surgeries required, hypotony less than 4 mmHg and visual loss. *EXG* exfoliation glaucoma, *FB* fornix-based, *IOP* intraocular pressure, *LB* limbal-based. *P < 0.05, **P < 0.01

**Table 3:** *P* values calculated by multivariable analysis using a Cox proportional hazards model for the surgical success

Parameter	Complete success^a^			Qualified success^a^		
	< 15 mmHg	< 18 mmHg	< 21 mmHg	< 15 mmHg	< 18 mmHg	< 21 mmHg
Width of filtration openings	–	0.0105^*^	–	0.0081**	0.0134*	–
Preoperative IOP	–	0.0512	–	0.0157*	0.0345*	–

^a^Surgical failure was defined as an IOP value ≥21 mmHg, ≥ 18 mmHg, or ≥ 15 mmHg with (qualified success) or without (complete success) the use of topical ocular hypotensive medication(s). Complete failure was defined as additional glaucoma surgeries required, hypotony less than 4 mmHg and visual loss. Parameters which had potential to be prognostic factor by univariable analysis (*P* < 0.10) were examined. *IOP* intraocular pressure. *P < 0.05, **P < 0.01

**Fig. 2:**
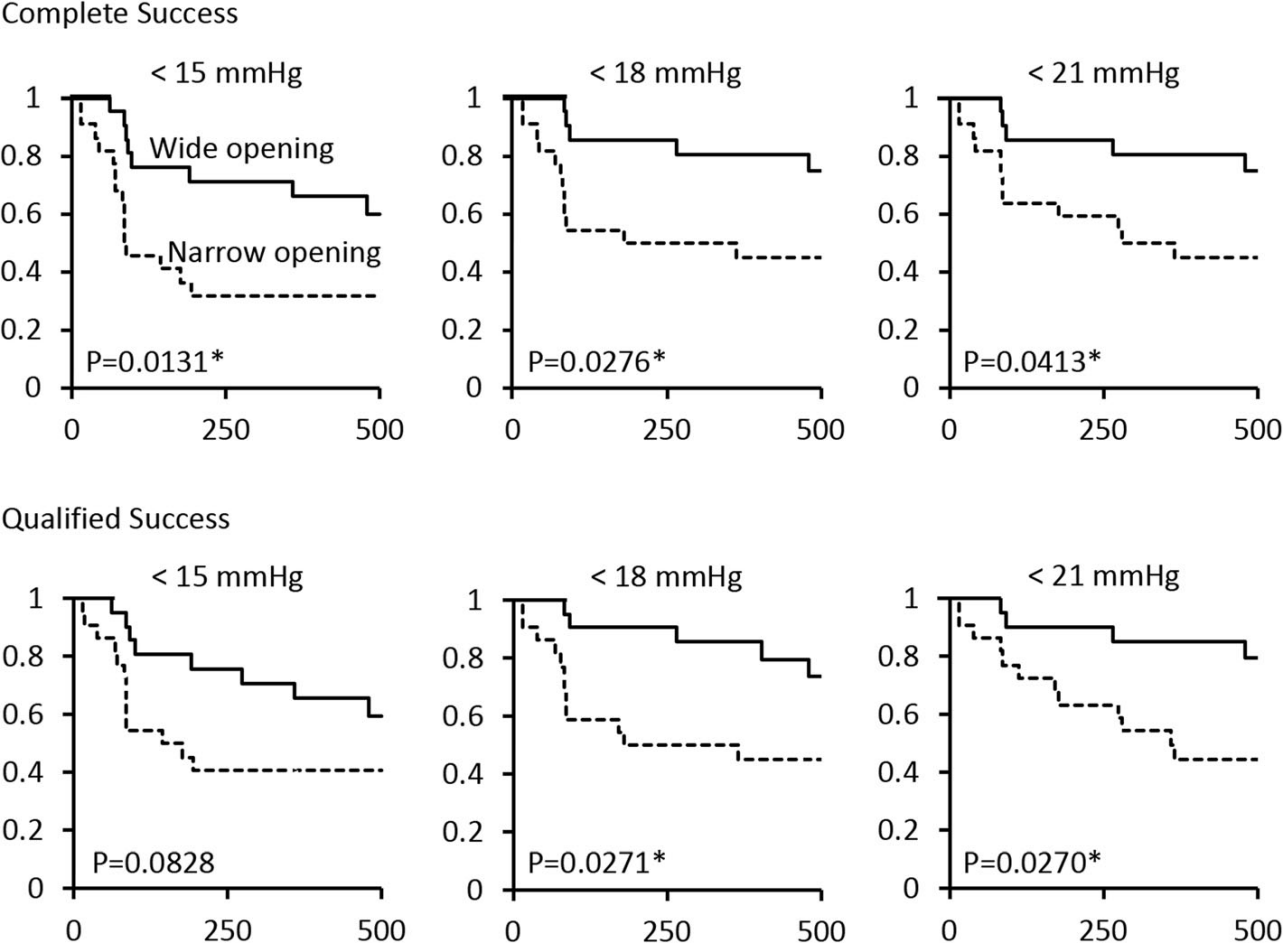
Kaplan-Meier survival plots of groups according to the width of the filtration openings on the scleral flap: eyes with filtration opening of > 1.78 mm, the median value of the patients (22 patients, solid line), and eyes with filtration opening of = < 1.78 mm (23 patients, dotted line). The y-axis indicates the success ratio (%), and the x-axis indicates the number of days after trabeculectomy

**Fig. 3:**
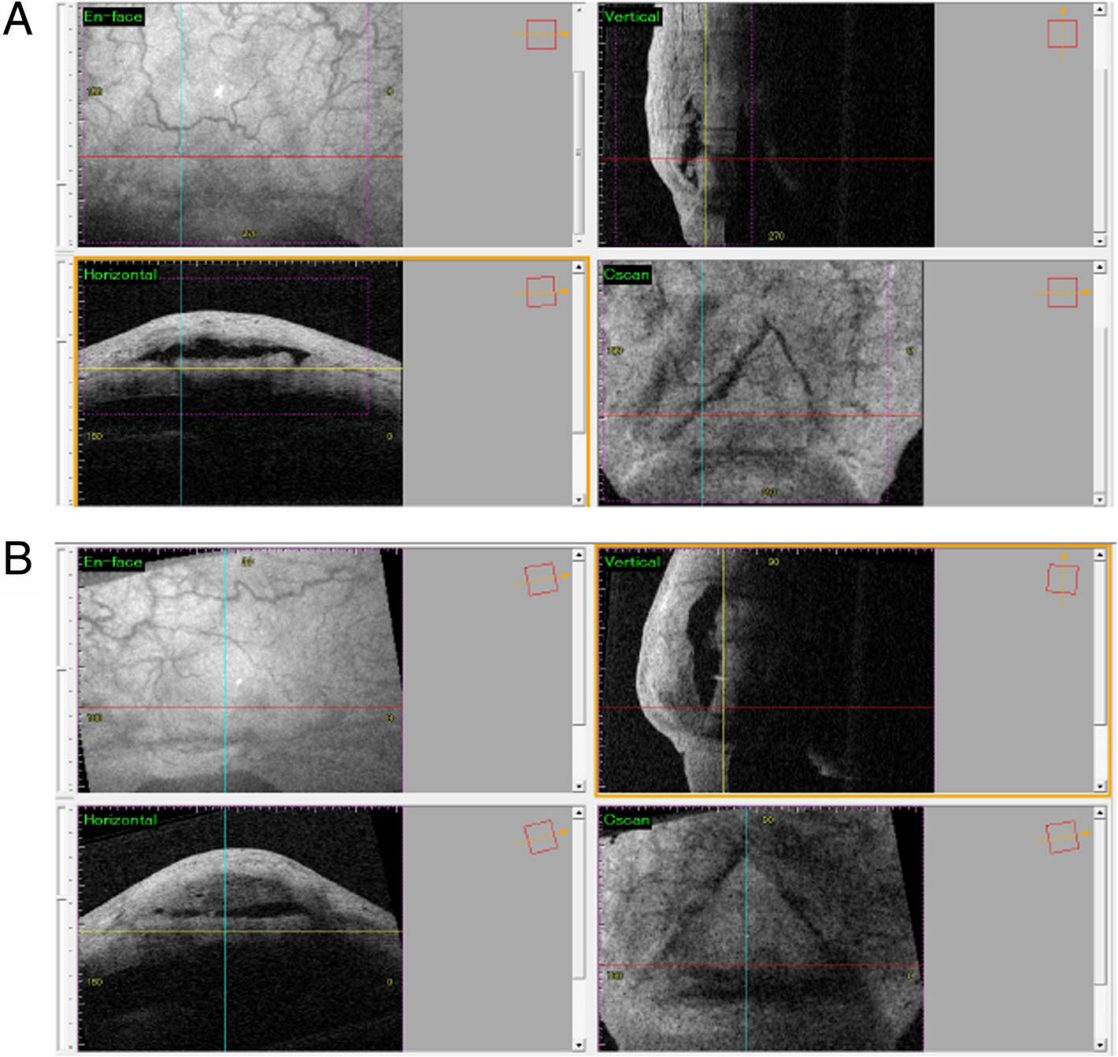
**a** Typical OCT images of the successful bleb with wide filtration opening at 2 weeks after trabeculectomy. The red and blue lines represent the horizontal and vertical axes, respectively. The yellow line indicates the z-axis of the data for C-scan images. The cross points of the colored lines show filtration openings, that continued to neighboring horizontal sections (not shown). IOP was 10 mmHg at 1 year after trabeculectomy without additional surgery or medication. **b** Typical OCT images of the failing bleb with no detectable filtration opening at 2 weeks after trabeculectomy. This case required needling bleb revision at 2 weeks after trabeculectomy

Then we subdivided the cases into two groups: those with wide filtration openings (greater than the median width of filtration openings, 1.78 mm; *n* = 22) and those with narrow filtration openings (= < 1.78 mm; *n* = 23). Kaplan-Meier survival analysis and Wilcoxon tests showed significant differences in success probabilities between the two groups for qualified success in criteria A and B and for complete success in all criteria, respectively (Fig. 2). The numbers at risk in the survival analysis are shown in Table 4. Typical images of the successful bleb with wide filtration opening and the failing bleb with narrow opening are shown (Fig. 3).

**Table 4:** Number at risk by time in Kaplan-Meier survival analysis

Time (day)	0	100	200	300	400	500
Wide opening	23	16	15	12	9	8
Narrow opening	22	20	19	18	16	13

## Discussion

In the previous study, the width of filtration opening at 0.5 months after trabeculectomy was inversely-correlated with the IOP value at 12 months after the operation [13]. In the present study, the long term IOP was also related with the width of filtration opening at 0.5 months after trabeculectomy. On the other hand, other bleb parameters including the thickness and intensity of the bleb wall did not show the relationship with the long term IOP control. That was contradicted with the previous knowledge conducted by OCT. [8, 15] The disaggregation might be given by the poverty of sample numbers in the present study. However, in spite of the limitation of the number or the observation periods, the significant correlation of the widths of filtration opening and long-term IOP is of great interest. Thus, the width of filtration opening might be more powerful than other bleb parameters to predict surgical success. We may be able to predict surgical success by assessing bleb by 3D AS-OCT.

In the present study, preoperative IOP was an independent prognostic factor for long term surgical success. In agreement with the present study, a previous prospective cohort study revealed that preoperative higher IOP was a risk factor for trabeculectomy success [16]. Based on these findings, glaucoma eyes with higher IOPs resistant to topical treatment may be more prone to excessive wound healing after filtration surgery. This may be due to presence of cytokines in the aqueous humor that have been associated with both higher IOPs and excessive wound healing. Of note, the concentration of aqueous MCP-1 tended to be high in refractory glaucoma types, including neovascular glaucoma and uveitic glaucoma [17–19], and was related to the width of filtration openings [13]. Indeed, the MCP-1 concentration in aqueous humor was a prognostic factor in open angle glaucoma [20].

In the past studies, the findings in the AS-OCT images of bleb wall were correlated to the IOP control [8, 9, 15]. Napoli et al retrospectively examined 20 filtering blebs using 2 types of OCTs working at a wavelength of 840 and 1310 nm, and found a significant association between good functionality and cystoid type at that time with both devices. In this study, the period between surgery and AS-OCT examination ranged from 3 months to 2 years [8]. These results may agree with the results of our past prospective study, presenting that the high-intensity bleb wall at 12 months post-trabeculectomy reflected the IOP and bleb wall vascularity at that time [21]. On the other hand, Nakano et al prospectively examined 48 eyes, and reported that blebs with uniform reflectivity at 2 weeks were significantly more likely to have worse function at 6 months [9]. In addition, Narita et al retrospectively assessed 99 eyes, and found that the striping phenomenon of the bleb wall at 2 weeks post-trabeculectomy was significantly associated with success at 1 year post-trabeculectomy [15]. These results suggest that the early bleb wall condition is a predictive factor of surgical success. Though bleb wall thickness or intensity did not relate to later IOP control in the present study, further large-scale prospective studies may clarify the relationship between early bleb wall condition and later IOP control.

The limitations of the present study were a relatively small sample size (45 eyes) and the retrospective study design. Additionally, the follow-up period (37 months in average) may not be enough to predict long-term outcomes, because the progression of wound healing after trabeculectomy is chronic, extending for years in some cases. Thus, we should interpret the data with caution. Prospective larger-scale studies with a longer follow-up period are essential.

## Conclusion

The width of filtration opening at an early stage may be a prognostic factor for surgical success of trabeculectomy in eyes with open-angle glaucoma.

## Data Availability

The datasets used and/or analyzed during the current study available from the corresponding author on reasonable request.

## Abbreviations

3D AS-OCT: Three-dimensional anterior segment optical coherence tomography
IOP: Intraocular pressure
OCT: Optical coherence tomography
UBM: Ultrasound biomicroscopy
